# Neuronal function of the mRNA decapping complex determines survival of *Caenorhabditis elegans* at high temperature through temporal regulation of heterochronic gene expression

**DOI:** 10.1098/rsob.160313

**Published:** 2017-03-01

**Authors:** Fivos Borbolis, Christina-Maria Flessa, Fani Roumelioti, George Diallinas, Dimitrios J. Stravopodis, Popi Syntichaki

**Affiliations:** 1 Biomedical Research Foundation of the Academy of Athens, Center of Basic Research, Athens 11527, Greece; 2 Faculty of Biology, School of Science, University of Athens, Athens, Greece; 3 School of Medicine, University of Athens, Athens, Greece

**Keywords:** *Caenorhabditis elegans*, *dcap-1*, *lin-14*, stress, developmental arrest

## Abstract

In response to adverse environmental cues, *Caenorhabditis elegans* larvae can temporarily arrest development at the second moult and form dauers, a diapause stage that allows for long-term survival. This process is largely regulated by certain evolutionarily conserved signal transduction pathways, but it is also affected by miRNA-mediated post-transcriptional control of gene expression. The 5′–3′ mRNA decay mechanism contributes to miRNA-mediated silencing of target mRNAs in many organisms but how it affects developmental decisions during normal or stress conditions is largely unknown. Here, we show that loss of the mRNA decapping complex activity acting in the 5′–3′ mRNA decay pathway inhibits dauer formation at the stressful high temperature of 27.5°C, and instead promotes early developmental arrest. Our genetic data suggest that this arrest phenotype correlates with dysregulation of heterochronic gene expression and an aberrant stabilization of *lin-14* mRNA at early larval stages. Restoration of neuronal *dcap-1* activity was sufficient to rescue growth phenotypes of *dcap-1* mutants at both high and normal temperatures, implying the involvement of common developmental timing mechanisms. Our work unveils the crucial role of 5′–3′ mRNA degradation in proper regulation of heterochronic gene expression programmes, which proved to be essential for survival under stressful conditions.

## 1. Introduction

Under adverse environmental conditions, survival of an organism depends on the induction of specific developmental programmes able to arrest metabolism and growth in a reversible manner. In *Caenorhabditis elegans* larvae, the dauer stage constitutes such an alternative developmental programme, elicited by harsh environmental conditions, such as nutrient limitation, overcrowding or high temperature [1]. Dauer larvae are animals arrested at a developmental stage equivalent to the second-to-third larval stage (L2-to-L3) moult, displaying extreme stress resistance and remarkably long lifespan. They can survive up to six months without feeding, whereas in favourable environments, they can resume growth and develop to reproductive adults with normal lifespan [2]. Identification and molecular characterization of *da*uer-*f*ormation (Daf) mutants has helped in defining the pathways that underlie dauer formation, including those of guanylyl cyclase, transforming growth factor-β (TGF-β), insulin/insulin-like growth factor (IGF-1) and steroid hormones [3]. These evolutionarily conserved signalling pathways act predominantly in the nervous system of worms to shape the decision between reproductive growth and dauer formation. Sensory neurons in particular greatly affect the decision of entering the dauer stage, as they receive and relay environmental cues to influence the aforementioned pathways [4].

Accumulating evidence demonstrates that temporal regulation of gene expression by post-transcriptional mechanisms plays an important role in cell fate decisions, developmental transitions and biological robustness in response to changing environments [5,6]. MicroRNAs (miRNAs) have been shown to control developmental events and maintain homeostasis by exerting repression on numerous target genes and providing speed, reversibility and compartmentalization of their actions [7]. Moreover, the presence of redundant components and regulatory loops ensures robustness against genetic or environmental perturbations [8]. For these reasons, some biological actions of individual miRNAs may be revealed only under specific developmental, genetic or environmental conditions [9–12]. *Caenorhabditis elegans* miRNAs were originally identified as components of the heterochronic pathway that controls post-embryonic developmental timing under normal conditions [13–15], whereas several miRNAs have been linked to dauer formation and stress condition management [16–18]. During the first larval stage (L1), accumulation of *lin-4* miRNA represses its heterochronic gene targets *lin-14* and *lin-28*, which encode a transcription factor and a cytoplasmic RNA-binding protein, respectively, thus allowing progression to later larval stages [19]. Continued expression of *lin-14* and *lin-28* at late stages, owing to loss of *lin-4*-mediated repression, was shown to result in retarded developmental events [20,21].

Regarding the mechanisms of miRNA-mediated gene silencing, several studies in developing worms have provided evidence for both translational inhibition and mRNA destabilization of *lin-14* and *lin-28* by *lin-4* miRNA [15,22–27]. Similarly, mammalian miRNAs have been shown to regulate protein levels by repressing translation and/or inducing degradation of target mRNAs [28–30]. The relative contribution of each molecular event to the overall gene silencing seems to be variable, depending on the specific miRNA, the cellular context and the environmental conditions [31–33]. Destabilization of mRNA targets is catalysed by enzymes involved in the 5′–3′ mRNA decay pathway, where mRNAs are first deadenylated, then decapped by a holoenzyme comprised the decapping proteins DCP1 and DCP2 (DCAP-1 and DCAP-2 in *C. elegans*), and finally degraded by the 5′–3′ exoribonuclease XRN1 [30]. Factors involved in the 5′–3′ mRNA decay pathway are often localized to evolutionarily conserved cytoplasmic RNA granules termed P-bodies, regulating mRNA turnover and translation, and possibly facilitating adaptation of cells to stress [34]. Furthermore, components of the mRNA decay and miRNA pathways have been observed in P-bodies and related RNA granules in neuronal cells, suggesting a role in local translation, which is critical for synapse development and plasticity [35]. In *C. elegans*, several studies of mutant or transgenic worms for decapping genes, have shown the important roles of the latter in diverse physiological processes including development, reproduction, stress response and ageing [36–40].

In this work, we have uncovered an essential role of the decapping complex in promoting early developmental decisions in worms under elevated temperatures. Mutations in decapping genes, although they enhance dauer formation of dauer-constitutive mutants at permissive temperatures, were shown to prevent entry of wild-type worms in the dauer stage at 27.5°C, inducing a prior and irreversible developmental arrest that precludes survival of the organism. This arrest phenotype of decapping mutants is mainly independent of the major dauer signalling pathways, although it is partially suppressed by loss of *daf-16*. Our tissue-specific rescuing analysis of *dcap-1* uncovered that loss of decapping activity, selectively in neurons, inhibits dauer formation at 27.5°C and impedes normal development at 25°C, at least in part through dysregulation of *lin-14* mRNA levels. In support of this, *lin-14(RNAi)* specifically in neurons could alleviate larval arrest of *dcap-1* mutants at high temperature. Moreover, we showed that the temporal control of a reporter gene by the *lin-14* 3′ untranslated region (3′UTR), where *lin-4* miRNA binds, was impaired in the neurons of decapping mutants at both temperatures. Consequently, our study unveils a specific role of the mRNA decapping complex in ensuring robust execution of heterochronic gene expression programmes upon environmental perturbations, with a great impact on organismal physiology and survival.

## 2. Results

### 2.1. Decapping mutants are prone to developmental arrest

**Figure 1.**
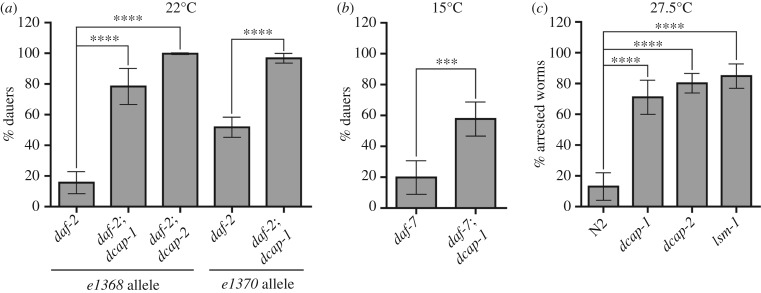
Disruption of *dcap-1* or *dcap-2* promotes developmental arrest. (*a*) Percentage of dauer arrested animals with defective insulin signalling at the permissive temperature of 22°C, after 3 days of development. (*b*) Percentage of dauer arrested animals with defective TGF-β signalling at the permissive temperature of 15°C, after 5 days of development. (*c*) Percentage of arrested animals with defective decapping activity at 27.5°C, after 3 days of development. The percentage of N2 animals corresponds to dauer arrest while in *dcap-1*, *dcap-2* and *lsm-1* animals it corresponds to L2-like larval arrest. Error bars represent standard deviation.

Mutations in *daf-2*, the single *C. elegans* insulin receptor orthologue, dampen transduction through the insulin/IGF-1 signalling (IIS) pathway and promote dauer formation during early development, or longevity and stress resistance in adulthood [41]. Previously, we have shown that disruption of *dcap-1* or *dcap-2* genes, encoding the subunits of the mRNA decapping complex in worms, significantly shortens the extremely long life of class I *daf-2(1368)* mutants and reduces their resistance to stress [39]. Intriguingly though, the double mutant *daf-2(e1368);dcap-1(tm3163)* or *daf-2(e1368);dcap-2(ok2023)* animals were found to display increased levels of dauer arrest at the permissive temperature of 22°C compared with *daf-2* single mutants (figure 1*a*). This was also true for the class II *daf-2(e1370)* hypomorphic allele (figure 1*a*). The *dcap-1(tm3163)* phenotype proved to be less penetrant than the null *dcap-2(ok2023)* owing to an out-of-frame deletion that completely eliminates *dcap-1* activity at 25°C, but retains a weak residual activity at lower temperatures [39]. At 22°C or 25°C, single decapping mutants did not form any dauers on their own, in the presence of food. Furthermore, we observed that the *dcap-1* mutation greatly enhances dauer arrest of *daf-7(e1372)* mutants at the permissive temperature of 15°C, forming approximately 65% dauers versus approximately 20% of the single *daf-7(e1372)* mutants (figure 1*b*). *daf-7* encodes a TGF-β-related ligand of the homonymous signalling pathway, which seems to regulate dauer formation in parallel to the IIS pathway [42]. Thus, loss of decapping activity sensitizes dauer-induced mutants and seems to influence specific developmental decisions.

These results prompted us to test whether loss-of-function (lf) of the decapping genes induces dauer formation at high temperatures, known to stimulate dauer entry independently of food or pheromones [43]. Wild-type (N2) animals, incubated at 27°C, form transient dauers in a small proportion (less than 20%) that is highly sensitive to subtle variations in environmental conditions [43]. In addition, several mutants in the genetic pathways regulating dauer formation with a weak or no dauer-constitutive phenotype at 25°C can enter the dauer stage at high penetrance at 27°C (a phenotype called Hid, for high-temperature-induced dauer formation) [43,44]. Therefore, we assayed dauer formation at a slightly higher temperature (27.5°C), where N2 formed dauers in a fraction ranging from 2% to 26%, as scored 3 days after egg transfer at this temperature (figure 1*c*). We observed that *dcap-1(tm3163)* or *dcap-2(ok2023)* mutants under these conditions exhibit a major arrest (ranging from 70% to 100%, figure 1*c*) at a persistently early developmental stage, morphologically similar to L2 larvae (electronic supplementary material, figure S1*a*). The *dcap-1* and *dcap-2* arrested animals were not true dauers as they were totally sensitive to 1% SDS (electronic supplementary material, figure S1*b*), did not induce a *sod-3::gfp* reporter at 27.5°C as dauers do, albeit there is some induction compared with N2 animals at this temperature (electronic supplementary material, figure S1*c,d*), and were dead by day 5 to 7. When 3-day arrested decapping larvae were transferred from 27.5°C to 15°C, only approximately 50% of *dcap-1* and approximately 30% of *dcap-2* were able to resume development to nearly sterile adults, in contrast to N2 dauers that fully recovered to fertile adults (electronic supplementary material, figure S2). Furthermore, we have observed high-temperature-induced larval arrest in *lsm-1(tm3585)* mutant animals (figure 1*c*), carrying a deletion in a cytoplasmic member of the Sm-like (LSm) protein family, which acts as a decapping activator [45,46]. Taken together, the above-mentioned results demonstrate that decapping mutants at 27.5°C exhibit an early arrest phenotype, without entering the dauer stage. Because this does not allow survival of the population until conditions improve and it is largely irreversible, we conclude that the decapping complex activity is crucial for survival of the organism in response to high temperature.

### 2.2. Loss of decapping activity induces larval arrest at high temperature independently of dauer signalling pathways

**Figure 2.**
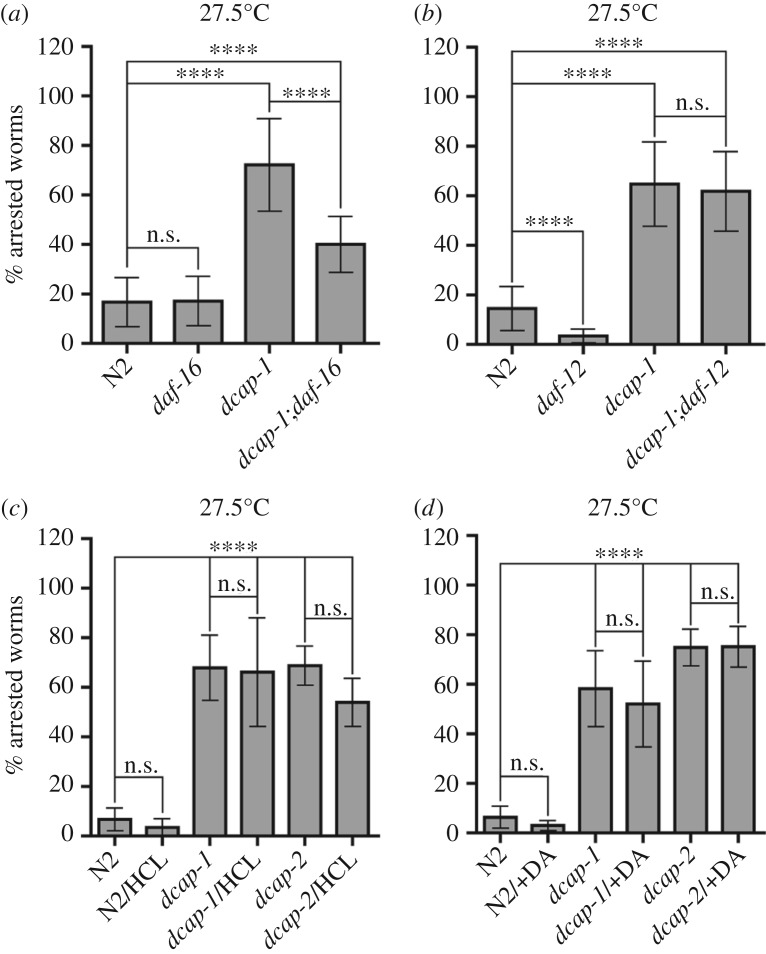
Loss of decapping activity induces larval arrest at a high temperature independently of dauer signalling pathways. Graphs showing the proportion of arrested animals at 27.5°C after 3 days of development (*a*) in *daf-16(mu86)* mutant background, (*b*) in *daf-12(rh62rh157)* mutant background, (*c*) supplemented with high cholesterol and (*d*) supplemented with DA. The percentage of N2, *daf-16* and *daf-12* animals corresponds to dauer or partial dauer arrest, while in *dcap-1*, *dcap-2* and double mutant animals it corresponds to L2-like larval arrest. HCL, high cholesterol (25 µg ml^−1^), DA, dafachronic acid (150 nM). Error bars represent standard deviation.

Developmental arrest of decapping mutants at high temperature could reflect defects of or variations in the signalling pathways that influence the choice to arrest in the dauer stage or proceed to reproductive growth in response to environmental signals [4]. Sensory neurons detect and integrate such signals to initiate the dauer programme through reduced production of cyclic guanosine monophosphate (cGMP) and downregulation of both the IIS and TGF-β signalling pathways. This, in turn, promotes dauer formation through unliganded nuclear hormone receptor DAF-12/NHR. The arrest phenotype of most known dauer-constitutive as well as Hid mutants fully depends on DAF-16/FOXO and DAF-12/NHR transcription factors [43,44,47]. In contrast, epistasis analysis of the double *dcap-1(tm3163);daf-16(mu86)* mutant that we generated using the null allele *daf-16(mu86)* [48] showed that loss of DAF-16 can only partially avert the arrest phenotype of the *dcap-1*, forcing development to the adult stage (figure 2*a*). Moreover, when we used the null allele *daf-12*(*rh62rh157)*, which is defective in dauer formation [49], to create *dcap-1;daf-12* double mutant animals, we observed that loss of *daf-12* did not suppress the number of arrested *dcap-1* larvae at 27.5°C (figure 2*b*).

The nuclear hormone receptor DAF-12/NHR functions downstream of the IIS and TGF-β signalling pathways to promote reproductive growth under favourable conditions, through the binding of its ligands, known as dafachronic acids (DA) [50]. Thus, we tested whether the incapability of decapping mutants to proceed to the adult stage at 27.5°C is attributed to low DAF-12 activation. As shown in figure 2*c,d*, high levels of cholesterol, which serves as a precursor of DA, or exogenous supplementation with DA in the culture medium, were not able to suppress the high percentage of arrested *dcap-1* or *dcap-2* worms and promote development. Hence, decapping mutations induce larval arrest at 27.5°C largely independently of the major dauer-signalling pathways, implicating defects in other developmental mechanisms. We hypothesize that such defects could be relieved to some extent upon disruption of *daf-16*, owing to the resulting global reprogramming of gene expression, as has been proposed for the *daf-16* mutants, which fail to arrest cell division during ‘L1 arrest’ under starvation [51].

### 2.3. Induction of the larval arrest phenotype of decapping mutants at high temperature is due to dysregulation of the heterochronic gene *lin-14*

**Figure 3.**
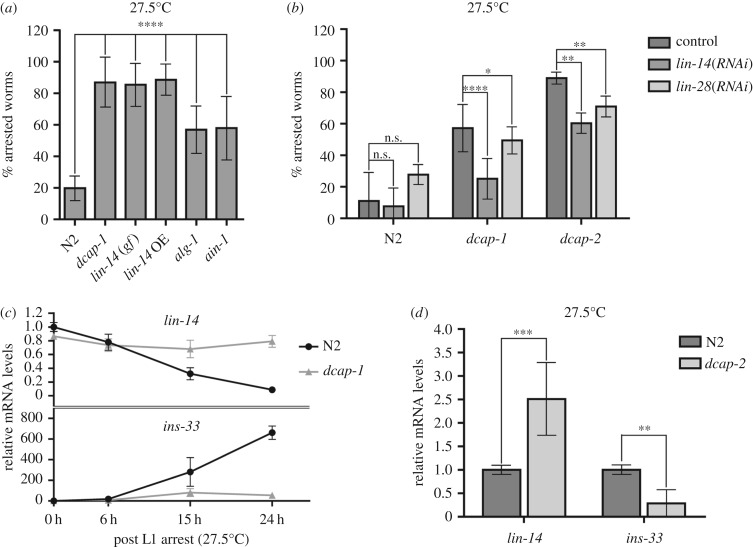
High LIN-14 levels control the larval arrest phenotype of decapping mutants at 27.5°C. (*a*) Percentage of arrested worms with high LIN-14 levels due to a gain-of-function (*gf*) mutation or overexpression (OE) of a *lin-14::gfp* transgene, and defective miRNA activity at 27.5°C, after 3 days of development. The percentage of N2 animals corresponds to dauer arrest, whereas in all other strains it corresponds to L2-like arrest. (*b*) Percentage of N2, *dcap-1* and *dcap-2* arrested animals fed with bacteria expressing *lin-14* or *lin-28* dsRNA at 27.5°C, after 3 days of development. (*c*) Time course analysis of *lin-14* and *ins-33* mRNA levels in N2 and *dcap-1* mutant animals grown at 27.5°C after plating L1 starved larvae (the hours correspond to time after release from L1 diapause). (*d*) Relative mRNA levels of *lin-14* and *ins-33* in N2 and *dcap-2* animals, 41 and 44 h respectively post-egg-laying at 27.5°C. Error bars represent standard deviation.

Progression through the larval stages in *C. elegans* is achieved through a heterochronic gene network, including specific transcription factors and the miRNAs that control their expression. Protein levels of LIN-14 transcription factor, which are temporally downregulated by *lin-4* miRNA, allow progression from L1 to L2 larval stage, whereas further downregulation at the end of L2 controls the transition to L3-specific gene expression [5,19]. LIN-14 levels can also specify the developmental stage at which dauer larva formation occurs in high density and in starved cultures [17]. Under such conditions, lf mutations in *lin-14* promote dauer entry at the L1 instead of L2 stage, whereas loss of *lin-4* miRNA or semi-dominant gain-of-function (gf) mutations in *lin-14* inhibit dauer formation upon starvation [17]. A *lin-14(n355gf)* mutation that removes all of the seven putative *lin-4* binding sites in the *lin-14* 3′UTR causes sustained expression of LIN-14 protein at stages later than the L1, reiterating L1 and L2 lineages [52]. When we exposed *lin-14(gf)* worms (strain MT355) to the temperature of 27.5°C, we observed an increased larval arrest similar to the one of decapping mutants (figure 3*a*). In addition, wild-type worms that have integrated a high-copy *lin-14::gfp* transgene (strain CT21) recapitulated the decapping mutant phenotype at 27.5°C (figure 3*a*, *lin-14* OE). Imaging analysis of LIN-14::GFP in wild-type (strain CT21) and *dcap-1* mutant background (strain BRF542) revealed that the transgene was similarly expressed at high levels during the first larval stage at 27.5°C (24 h post-egg-hatching), but on the second day, although the GFP signal was decreased in L2 wild-type worms, it was still bright in *dcap-1* mutants (electronic supplementary material, figure S3). Because miRNA-induced destabilization of mRNA targets is catalysed by enzymes involved in the 5′-3′ mRNA decay pathway, we presume that high levels of LIN-14 owing to impaired degradation of its mRNA in the decapping mutants, could interfere with the developmental decision that needs to be made by early larvae at high temperature, in order to enter the dauer stage or proceed to later developmental stages.

In support of this hypothesis, loss of *alg-1*, encoding an argonaute-like protein with a role in the maturation of miRNAs and their function as part of the microRNA-induced silencing complex (RISC) [53,54], promotes to some extent larval arrest of otherwise wild-type worms at 27.5°C (figure 3*a*). Similarly, enhanced larval arrest was scored upon deletion of *ain-1*, encoding a GW182 homologue that interacts with argonaute proteins to form RISC complexes and enhance miRNA activity by targeting repressed mRNAs for transfer and degradation into P-bodies [23,55,56] (figure 3*a*). More importantly, knockdown of *lin-14* mRNA by RNA interference (RNAi) greatly reduced the percentage of *dcap-1* or *dcap-2* arrested larvae (figure 3*b*), while a smaller but significant reduction in the number of decapping arrested larvae was observed upon *lin-28(RNAi)* (figure 3*b*). LIN-28 is an RNA-binding protein that is highly expressed in L1 stage and positively regulates LIN-14 protein levels independently of *lin-4* [20]. As shown recently, LIN-28 binds to the 3′ UTR of *lin-14* mRNA and stabilizes it, whereas loss of *lin-28* reduces *lin-14* mRNA levels [57]. To determine whether *lin-14* does in fact accumulate in *dcap-1* mutants grown at 27.5°C, we measured *lin-14* mRNA levels in total RNA extracts isolated from synchronized N2 and *dcap-1* larvae, after plating arrested L1 hatchlings on NGM plates seeded with bacteria and collecting worms at 0, 6, 15 and 24 h of post-embryonic development at high temperature. We observed an intense stabilization of *lin-14* mRNA levels in *dcap-1* worms at all time-points, compared with N2, in which the mRNA levels were found to progressively decrease (figure 3*c*). Likewise, *lin-14* mRNA levels were substantially increased in *dcap-2* L2 larvae compared with similarly staged N2 animals, collected 44 and 41 h post-egg-hatching at 27.5°C, respectively (figure 3*d*). In addition, *ins-33* transcripts were shown to be downregulated in *dcap-1* and *dcap-2* versus N2 worms (figure 3*c*,*d*), which is consistent with the fact that *ins-33* (an insulin-like peptide) is a directly downregulated target of LIN-14 in N2 larvae [58].

**Figure 4.**
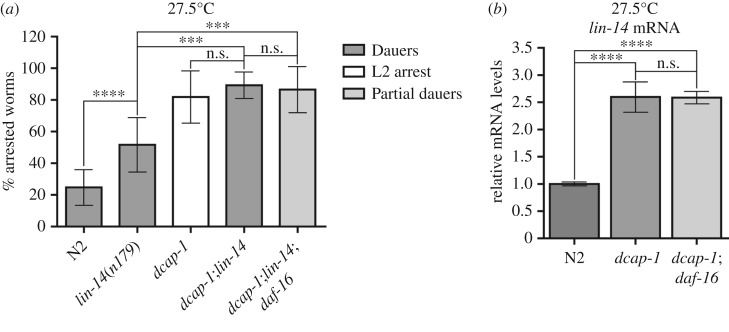
*lin-14* accumulates independently of DAF-16 in decapping mutants and acts downstream of *dcap-1* to drive developmental arrest. (*a*) Percentage of arrested *dcap-1*;*lin-14* and *dcap-1;lin-14;daf-16* animals in 27.5°C after 3 days of development. The percentage of N2, *lin-14* and *dcap-1;lin-14* corresponds to dauer arrest, of *dcap-1;lin-14;daf-16* to incomplete dauer arrest and of *dcap-1* in L2-like arrest. (*b*) Relative mRNA levels of *lin-14* in N2 animals at 44 h and in *dcap-1* and *dcap-1;daf-16* animals at 48 h post-egg-laying in 27.5°C. Error bars represent standard deviation.

Overall, *lin-14* mRNA levels accumulate in decapping mutants and knockdown of *lin-14* by RNAi greatly reduces their arrest phenotype at 27.5°C. To further examine whether loss of *lin-14* is sufficient to completely suppress the decapping phenotype, we used a temperature-sensitive *lin-14* allele, *n179*, which is a weak hypomorphic allele at the permissive temperature of 15°C but exhibits *lin-14* loss-of-function phenotypes at 25°C [52,59]. We observed that *dcap-1(tm3163);lin-14(n179)* double mutants grown at 25°C displayed more severe *lin-14* heterochronic phenotypes (precocious development, semi-dumpy, vulval protrusions and egg-laying defects) than single *lin-14* mutants, while a few animals were arrested in early larval stages (mostly L1 and L2) resembling dauers in morphology. Interestingly, dauer arrest at both the L1 and L2 larval moult had been previously reported for several *lin-14(lf)* mutants in high-density and starved cultures, implying that *lin-14* activity plays a negative role in dauer initiation at these stages [17]. When we exposed *lin-14* mutants to the temperature of 27.5°C more than 50% of the animals were found to be arrested at these early larval stages (figure 4*a*), exhibiting dauer-specific features. Under the same conditions, approximately 90% of *dcap-1;lin-14* double mutants were early arrested and all of them resembled *lin-14* dauer larvae, regarding the stage of arrest and their morphology (highly refractile material in the intestine, constricted pharynxes and plugged mouth as shown in electronic supplementary material, figure S4). Thus, loss of *dcap-1* enhances the penetrance of weak *lin-14(n179)* allele while loss of *lin-14* fully suppresses the non-dauer phenotype of L2 arrested *dcap-1* mutants.

Because we have found that loss of DAF-16 function can partially suppress the decapping mutant phenotype, and DAF-16 is known to be required for L1 and L2 developmental arrest [41], we asked whether this dauer phenotype of *dcap-1;lin-14* double mutants depends on DAF-16. By constructing the *dcap-1(tm3163);lin-14(n179);daf-16(mu86)* triple mutant, we observed that loss of *daf-16* results in slightly more severe *lin-14* heterochronic phenotypes, when compared with *dcap-1;lin-14* double mutant, at all temperatures. Also, the percentage of early arrested larvae at 27.5°C in the triple mutant was similar to that of the *dcap-1;lin-14* (figure 4*a*), but these arrested animals have a more transparent gut and incomplete remodelling in the pharynx and mouth (electronic supplementary material, figure S4). Thus, our analysis suggests that DAF-16 is not required for developmental arrest of *dcap-1;lin-14* animals, although loss of *daf-16* affects the implementation of the committed transcriptional programme. Since DAF-16 activity has been shown to repress, possibly indirectly, the transcription of *lin-4* miRNA during L1 arrest under starvation [51], we tested the levels of *lin-14* mRNA in *dcap-1* and *dcap-1;daf-16* arrested larvae at 27.5°C, but we did not observe any change in the elevated levels of *lin-14* when *daf-16* is missing (figure 4*b*). This indicates that the partial suppression of *dcap-1* arrest phenotype by *daf-16* allele (figure 2*a*) does not correlate with reduced *lin-14* levels but rather with altered gene expression in *daf-16* background, forcing animals to escape arrest.

### 2.4. Restoration of *dcap-1* expression or downregulation of *lin-14* only in neurons promote development of *dcap-1* mutants at high temperature

**Figure 5.**
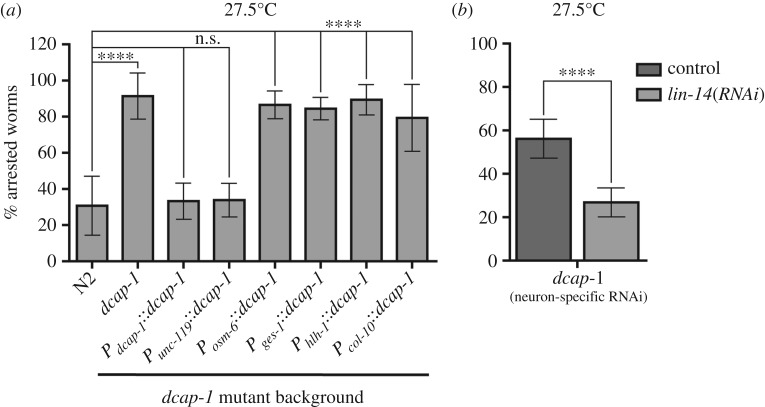
Neuron-specific *dcap-1* function influences developmental progression at high temperatures by regulating neuronal LIN-14 levels. Percentage of arrested *dcap-1* mutant animals (*a*) expressing a *dcap-1::gfp* transgene in selective tissues at 27.5°C (N2 and *dcap-1* controls carry a *rol-6(su1006)* transgene (rollers)) and (*b*) engineered for neuron-specific RNAi, fed with bacteria expressing *lin-14* dsRNA at 27.5°C. Error bars represent standard deviation.

In *C. elegans*, *lin-4* and its targets *lin-14* and *lin-28* are expressed in several cell types, such as hypodermis, muscles and neurons [20,21], resembling the expression pattern of *dcap-1* that we had previously determined using a translational *dcap-1::gfp* reporter [39]. To identify the major site of DCAP-1 function in developmental decisions at high temperature, we scored the percentage of arrested larvae in a series of transgenic animals expressing *dcap-1::gfp* in selective tissues. In this way, we revealed that ectopic expression of *dcap-1* in neurons only (using the *unc-119* promoter) was sufficient to fully suppress the arrest phenotype of *tm3163* at 27.5°C, similarly to *dcap-1* expression under the endogenous promoter (figure 5*a*). On the contrary, expression in the intestine (driven by the *ges-1* promoter), muscles (*hlh-1* promoter) or hypodermis (*col-10* promoter) failed to provide any rescue (figure 5*a*). Intriguingly, restoration of *dcap-1* function only in sensory neurons of *dcap-1* mutant (using the *osm-6* promoter) was not sufficient to complement the mutant phenotype (figure 5*a*).

Based on our findings that *lin-14* mRNA is greatly stabilized in *dcap-1* mutants and the expression of a pan-neuronal *dcap-1::gfp* transgene was able to rescue for their arrest at 27.5°C, we wondered whether suppression of the mutant phenotype upon *lin-14(RNAi)* was due to *lin-14* downregulation in neurons or in other tissues. Feeding RNAi in *C. elegans* is more effective in non-neuronal cell types [60], although selective sensitivity of neurons to RNAi has been described [61]. In order to establish the possible functional role of *lin-14* in neurons, we performed cell-autonomous RNAi specifically in neuronal cells. One approach that allows effective tissue-specific RNAi by feeding is the selective expression of a rescuing SID-1 dsRNA transporter in a systemic RNA interference deficient-1 (*sid-1*) mutant background, where systemic spread of silencing does not occur [62,63]. We generated transgenic *dcap-1(tm3163);sid-1(pk3321)* animals carrying the wild-type *sid-1* gene under the control of the pan-neuronal *unc-119* promoter (*P_unc-119_::sid-1*), along with two GFP reporters in neurons and body wall muscles (*P_unc-119_::gfp* and *P_myo-3_::gfp*) to assess cell-autonomous and systemic RNAi for *gfp*, concurrently. Transgenic animals displayed the *unc* (uncoordinated) phenotype in response to *unc-13(RNAi)* feeding, a known neuronal gene [63], whereas effective silencing of GFP in neurons but not in muscles, was verified upon feeding with *gfp(RNAi)* (data not shown). When these worms were fed with *lin-14* dsRNA, we observed a significant suppression of the arrest phenotype at 27.5°C (figure 5*b*). Thus, downregulation of *lin-14* expression only within the nervous system is able to promote development of *dcap-1* larvae at high temperature.

**Figure 6.**
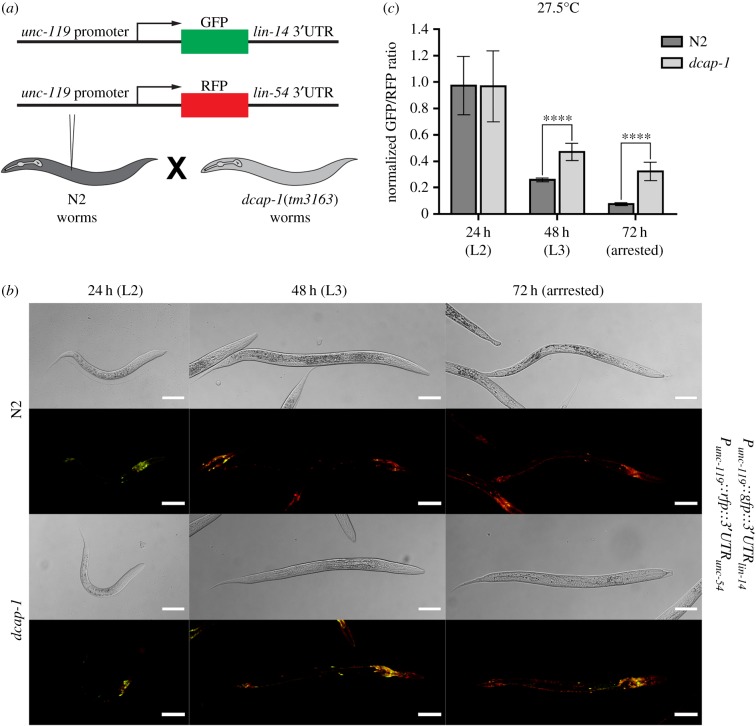
Misregulation of a *gfp* reporter bearing the *lin-14* 3′UTR in the nervous system of *dcap-1* mutants at 27.5°C. (*a*) Schematic of the construction of transgenic animals. Two different constructs, a *gfp* gene fused to *lin-14* 3′UTR and an *rfp* gene fused to *unc-54* 3′UTR under the control of the pan-neuronal promoter *unc-119*, were co-injected in N2 worms. These transgenic animals were then crossed with *dcap-1* mutant males. (*b*) Representative confocal images of N2 and *dcap-1* worms expressing the two translational reporters 24, 48 and 72 h post-egg-laying at 27.5°C. Scale bar , 50 µm. (*c*) Normalized ratio of GFP/RFP fluorescence 24, 48 and 72 h post-egg-laying at 27.5°C. Fluorescence was normalized to N2 animals at 24 h. Error bars represent standard deviation.

The *lin-14* 3′UTR is necessary and sufficient to confer *lin-4*-mediated silencing of a reporter gene [15]. Thus, we further examined whether the 3′UTR is important for regulation of *lin-14* expression levels in the nervous system of *dcap-1* worms. To this end, we created a *P_unc-119_::gfp* reporter fused to the *lin-14* 3′UTR element, and we expressed it in neurons along with a *P_unc-119_::rfp* reporter bearing a 3′UTR with no miRNA-binding sites (3′UTR of *unc-54*), which was used as a normalizing control (figure 6*a*). To exclude any possible discrepancies owing to mosaicism in transgenic worms, we quantified the ratio of GFP:RFP signal over time in neurons of several individual N2 and *dcap-1* worms grown at 27.5°C (figure 6*b*). The normalized fluorescence intensity ratio was similar in N2 and *dcap-1* animals at 24 h post-egg-laying (L2 stage; figure 6*c*). However, after 48 h (L3 stage), this ratio was found to be reduced by 74% in wild-type animals versus 51% in *dcap-1* arrested animals. Moreover, by quantifying the signal in N2 dauers and *dcap-1* arrested larvae after 72 h at 27.5°C, we observed a significant downregulation in the expression of *P_unc-119_::gfp::3′UTR_lin-14_* reporter in true dauers compared with *dcap-1* arrested animals (figure 6*b*,*c*). Hence, the *lin-14* 3′UTR-mediated regulation of gene expression is impaired in the nervous system of decapping mutants at high temperature.

### 2.5. Loss of function of decapping genes influences heterochronic gene expression and growth rates during normal development

**Figure 7.**
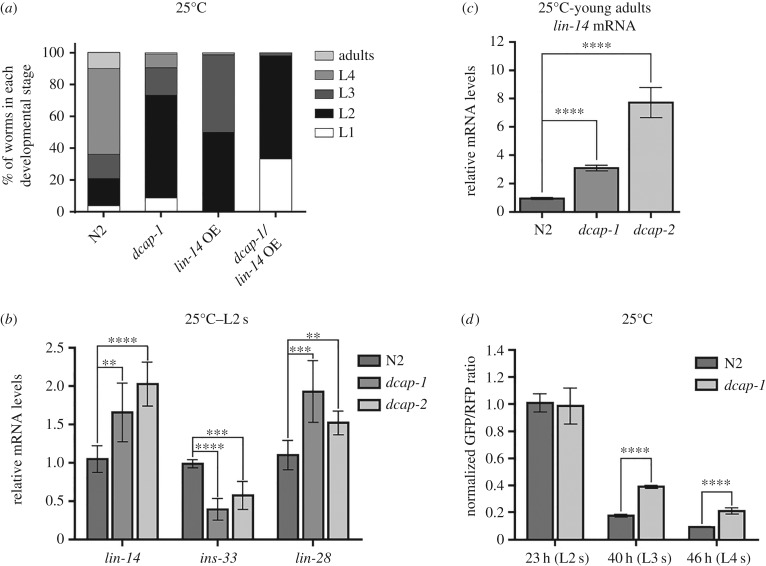
Decapping activity influences heterochronic gene expression and growth rate during normal development. (*a*) Distribution of N2 and *dcap-1* mutant animals overexpressing *lin-14::gfp* among developmental stages, 48 h post-egg-laying at 25°C. N2 and *dcap-1* controls carry a *rol-6(su1006)* transgene (rollers). (*b*) Relative mRNA levels of *lin-14*, *ins-33* and *lin-28* in L2-staged wild-type and decapping mutant animals, grown at 25°C. (*c*) Relative mRNA levels of *lin-14* in young adults of N2 and decapping mutants, grown at 25°C. (*d*) Normalized ratio of GFP/RFP fluorescence 23, 40 and 46 h post-egg-laying at 25°C, in N2 and *dcap-1* mutants carrying a *gfp::3′UTR_lin-14_* and an *rfp::3′UTR_unc-54_* transgene under the control of the pan-neuronal promoter *unc-119*. Fluorescence was normalized to N2 animals at 23 h. Error bars represent standard deviation.

During post-embryonic development of worms under favourable, non-stressful conditions, protein levels of LIN-14 are temporarily regulated by *lin-4* miRNA at the level of both mRNA translation and degradation [14,22,64]. Worms lacking *lin-4* or persistently expressing *lin-14* clearly display a retarded heterochronic phenotype compared with N2 [59], something that we also verified in the strain CT21 bearing a high-copy *lin-14::gfp* transgene (figure 7*a*). Moreover, we noted that this *lin-14::gfp* transgene further enhanced the slow development of *dcap-1* mutants, at 25°C (figure 7*a*). At this temperature, *dcap-1* as well as *dcap-2* and *lsm-1* mutants tend to be asynchronous, even after tightly synchronous egg-laying, whereas adults exhibit egg-laying defects, reduced fertility and uncoordinated movement [36,39]. Intrigued by the dysregulation of *lin-14* in decapping mutants in response to high temperature, we investigated whether the levels of *lin-14* mRNA are also affected in *dcap-1* mutant worms during normal development at 25°C. Thus, we tested the mRNA levels of *lin-14*, *lin-28* and *ins-33* in properly synchronized and morphologically similar (L2 stage) N2, *dcap-1* and *dcap-2* animals, grown at 25°C. In agreement with the above results, the mRNA levels of *lin-14* and *lin-28* were found to be upregulated in both decapping mutants, whereas the expression of *ins-33* was decreasing (figure 7*b*). Interestingly, when we measured the mRNA levels of *lin-14* in young adults from all strains, we observed a significant increase in decapping mutants, compared with wild-type animals (figure 7*c*). Therefore, it is plausible to speculate that the upregulation of *lin-14* mRNA levels during adulthood could contribute to or even account for the short lifespan of decapping mutants [36,39], given that *lin-4* and *lin-14* genes function to regulate ageing, with reduced *lin-14* activity extending the lifespan of worms [65].

Our analysis also revealed that only the native and neuronal *dcap-1::gfp* transgenes were able to rescue growth defects of *dcap-1(tm3163)* at the temperature of 25°C (electronic supplementary material, figure S5). Thus, we used the aforementioned neuronal GFP and RFP reporters to test whether the transgene bearing the *lin-14* 3′UTR is subject to dysregulation in neuronal cells of *dcap-1* mutants when they are grown at the normal temperature of 25°C. Indeed, we measured a substantial increase in the GFP : RFP ratio in *dcap-1* versus N2 worms over time at 25°C during early development (figure 7*d*; electronic supplementary material, figure S6). We also noted that *dcap-1* transgenic animals bearing this neuronal *P_unc-119_::gfp::3′UTR_lin-14_* reporter displayed a developmental delay compared with synchronized *dcap-1* worms expressing only the *rol-6* co-transformation marker. This finding implies that dysregulation of *lin-14* mRNA levels in decapping mutants owing to diminished degradation could affect growth through sequestration of *lin-4* miRNA molecules or other components of the miRNA pathway on *lin-14* 3′UTR. A similar effect of titration of *lin-4* activity has been described in animals overexpressing *lin-14* 3′UTR in hypodermal tissues, resulting in abnormal adult alae formation [66].

**Figure 8.**
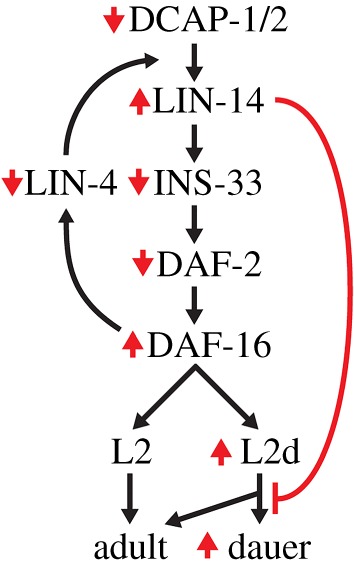
Integrative model for the molecular interactions mediating the effects of aberrant decapping in developmental arrest. Mutations in decapping genes lead to insufficient levels of *lin-4*-mediated silencing and higher levels of LIN-14 that have a negative impact in *ins-33* expression. Low levels of INS-33 might cause a fine decrease in insulin signalling, slightly elevating DAF-16 activity, thus sensitizing Daf-c worms to arrest their development as dauers at permissive temperatures. However, under elevated ambient temperature (27.5°C), high levels of LIN-14 in otherwise wild-type decapping mutants impair their ability to proceed to either adulthood or dauer diapause, leading to an irreversible developmental arrest at a stage that resembles the L2 (L2d) larva, independently of DAF-16 activity. Red arrows and line correspond to the effect of mutations in decapping genes.

From all the above, we infer that neuron-specific *dcap-1* function can influence developmental progression and decisions at both normal and high temperatures, at least in part through regulation of heterochronic gene expression. An integrative model for the molecular interactions mediating the effects of aberrant decapping in developmental arrest is presented in figure 8.

## 3. Discussion

Post-transcriptional control of gene expression is critical for determining the amounts of proteins present in eukaryotic cells. At the level of mRNA metabolism, this is achieved through mechanisms that regulate translation rate and mRNA turnover in the cytoplasm. One such mechanism in all eukaryotes is the 5′–3′ mRNA decay pathway that degrades deadenylated/oligoadenylated mRNAs by the action of XRN1 exoribonuclease, after decapping by the DCP1/DCP2 complex. Proper regulation of mRNA decay rate is critical for cells in order to maintain their homeostasis, through the degradation of no-longer-needed or aberrant transcripts that could lead to the production of harmful proteins. Moreover, the 5′–3′ mRNA decay pathway plays an important role in miRNA-mediated post-transcriptional control. miRNAs typically bind to sites in the 3′UTRs of their target mRNAs, in order to recruit the RISC complex, and thus inhibit translation and promote mRNA degradation through deadenylation, decapping and 5′–3′ decay [29]. From a functional point of view, miRNAs are either key regulators of spatio-temporal gene expression or ‘fine-tuners’ that help to provide robustness in nearly all biological processes [67], while offering a mechanism for rapid response to stress conditions [6]. Several paradigms support a model whereby miRNAs trigger the deadenylation and decay of target mRNAs during early development or in response to extracellular stimuli.

In *C. elegans*, mutants in components of the 5′–3′ mRNA decay pathway display pleiotropic phenotypes, revealing their importance in diverse physiological processes, such as development, reproduction, stress response and ageing [36,37,39,68–70]. However, there is little information so far about their influence on specific mRNAs, which lead to the observed phenotypes when dysregulated. Although the 5′–3′ mRNA decay pathway has a central role in the turnover of the bulk of mRNAs, gene-specific roles might be prominent in particular stages of development, as suggested by the function of several mRNA metabolism factors during oogenesis or early embryogenesis [68–70]. Likewise, the activity of those factors might be more critical under specific environmental conditions or in specific tissues. Here, we have shown that neuronal function of *dcap-1* is essential for the survival of worms at the high temperature of 27.5°C, owing to its impact on dauer formation. In this unfavourable environment, wild-type worms can enter the dauer stage at a low percentage, but decapping mutants fail to complete this transition and get trapped at an earlier stage. Neuronal expression of *dcap-1* is sufficient to fully rescue the arrest phenotype of mutants at 27.5°C, similar to endogenous promoter-driven *dcap-1* expression. Likewise, restoration of *dcap-1* function in neurons can rescue the developmental defects of *dcap-1* mutants at normal temperature (25°C), suggesting the existence of similar underlying molecular events that influence developmental decisions at both temperatures.

The nervous system is an important site of action for several regulatory pathways, which modulate organismal stress response in order to maintain systemic protein homeostasis [71]. Under unfavourable conditions, the IIS and TGF-β signalling pathways operate in the nervous system of worms to modulate dauer formation, along with unliganded DAF-12/NHR [4]. We have shown here that disruption of *daf-12* or, alternatively, induction of its activity by exogenous supplementation with steroid hormone does not cause any suppression of the *dcap-1* phenotype at 27.5°C. In contrast, loss of decapping activity in dauer-constitutive mutants of the IIS and TGF-β pathways greatly enhances dauer formation at permissive temperatures, in a DAF-16-dependent mode (data not shown). This could reflect deregulation of control mechanisms which normally preserve cellular homeostasis when the function of the above-mentioned signalling pathways is compromised. Interestingly, a role of neuronal miRNAs in preventing aberrant dauer formation has been shown, proposing miRNA-mediated gene silencing as a mechanism that reinforces the robustness of these pathways [18]. In addition, several regulators of developmental timing, including *lin-4* and the *let-7*-family miRNAs, along with their targets LIN-14, LIN-28, HBL-1 and DAF-12, can modulate dauer formation under adverse conditions [16,17,49,72], while also contributing to the robust developmental progression during post-dauer stages [73].

Consistent with a role of mRNA degradation in miRNAs' function, during larval development of worms, the levels of LIN-14 transcription factor are temporally regulated *by lin-4* miRNA at the level of both mRNA translation and stabilization, whereas the 5′–3′ exoribonuclease XRN-1 seems to have an important role in the decay of *lin-4* and *let-7* mRNA targets [14,22,23,25,27,64]. Analysis of *lin-14* mRNA and protein levels over the course of early larval development in wild-type worms has revealed a biphasic mode of regulation: a fast *lin-14* mRNA destabilization, as soon as *lin-4* miRNA is expressed in mid-L1 stage and a subsequent long-term translational inhibition with reduced LIN-14 protein levels and no obvious further decline in mRNA levels [26]. Here, in a time frame from 0 to 24 h post-embryonic development at 27.5°C, we have observed a similar early destabilization of *lin-14* mRNA in wild-type worms, but not in *dcap-1* mutants, where a persistent stabilization of mRNA was demonstrated. Therefore, we infer that stabilization of *lin-14* mRNA in decapping mutants during early development is inhibitory to the progression of normal developmental events at 27.5°C. In support of this, *lin-14(gf)* mutations that cause sustained expression of *lin-14* in later stages than the L1, inhibit dauer formation in high-density and starved cultures [17]. As we have shown, these *lin-14(gf)* animals were also unable to form dauers at 27.5°C and remained arrested in an earlier stage, similar to worms overexpressing a *lin-14::gfp* transgene or bearing deletions of core components of RISC. Moreover, downregulation of *lin-14* by feeding RNAi partially suppressed the arrest phenotype of decapping mutants at 27.5°C, forcing development to later stages.

In a *dcap-1;lin-14(lf)* double mutant, loss of *lin-14* causes an earlier arrest of animals in the L1 and L2 stages, with dauer-specific features, similar to the single *lin-14(lf)* mutant (figure 4*a*; electronic supplementary material, figure S4). The increased ratio of dauer arrest in double versus single mutant is consistent with the observed enhanced sensitivity of dauer-induced mutants in *dcap-1* background. Thus, in accordance to the negative role of LIN-14 in dauer initiation at L1 or L2 stage, in starved cultures [17], elimination of *lin-14* function fully suppresses the non-dauer phenotype of L2 arrested *dcap-1* mutants, restoring normal developmental progression to dauers. We suggest that in *lin-14(RNAi)*-treated *dcap-1* mutants progression to adults (exhibiting heterochronic phenotypes) is favoured against entrance in the dauer stage owing to partial downregulation of *lin-14* in arrested L2 animals. The arrest phenotype of *dcap-1* was also partially suppressed by the deletion of *daf-16* gene, in a double mutant *dcap-1;daf-16* (figure 2*a*). This effect could be attributed to regulation of LIN-14 levels by DAF-16, since DAF-16 activity has been found to repress, possibly indirectly, the transcription of *lin-4* miRNA during L1 arrest under starvation [51]. This functional interaction prompted us to assess the levels of *lin-14* mRNA in *dcap-1* and *dcap-1;daf-16* arrested larvae at 27.5°C, which revealed that no change in the elevated levels of *lin-14* occurs when *daf-16* is missing (figure 4*b*), supporting a model in which accumulation of *lin-14* mRNA in *dcap-1* mutants occurs independently of DAF-16. In addition, this result suggests that partial suppression of the *dcap-1* arrest phenotype by the mutant *daf-16* allele (figure 2*a*) does not correlate with reduced *lin-14* levels but rather with altered gene expression in *daf-16* background, forcing animals to escape arrest, an effect analogous to the promotion of post-embryonic development owing to loss of *daf-16* in arrested L1 larvae during starvation [51].

Our tissue-specific rescuing experiments of *dcap-1* suggest a close connection between decapping activity and temporal miRNA-regulated expression of *lin-14* in neuronal tissues. The functional role of this relationship in the nervous system is further highlighted by the observation that neuron-specific downregulation of *lin-14* is sufficient for the suppression of the arrest phenotype. In agreement with the above, *lin-14* 3′UTR-mediated regulation of gene expression was found to be impaired in the nervous system of decapping mutants at both high and normal temperatures. In *C. elegans*, *lin-4* miRNA regulates the timing of stage-specific cell division patterns in hypodermal, muscle and vulval lineages [5], but also the timing of synaptic remodelling and axon extension in neurons [24,74,75]. Likewise, a member of the *miR-125* family, the human homologues of *lin-4* in worms, has been involved in the regulation of dendritic spine morphology and synaptic transmission in mouse hippocampal neurons [76]. Thus, it is plausible that dysregulation of miRNA functions in the nervous system of decapping mutant worms controls growth and survival by causing alterations in neuronal development and synaptic function. In support of this, a mutation that impairs the deadenylation-dependent mRNA decay pathway in zebrafish, affects mRNA levels of developmental control genes that serve in the differentiation of dopaminergic neurons [77]. In many organisms, including mammals, components of the 5′–3′ mRNA decay and miRNA pathways have been observed in neuronal P-bodies or related RNA granules, suggesting a role for them in local mRNA translation. In line with this, miRNAs are especially abundant in neurons and their rapid decay has been linked to neuronal activity [78]. Thus, dysregulated mRNA localization and translation is connected to pathology of several neurodevelopmental and neurodegenerative diseases [35].

Several miRNAs, some of them evolutionarily conserved, have specific roles in neuronal development and function in *C. elegans* [67,79], but the extent to which the mRNA degradation machinery regulates their activity is currently unknown. Neuronal miRNA and mRNA decay mechanisms might also promote rapid stress responses and survival during environmental changes. A number of studies have shown that stress affects the expression levels of miRNAs as well as their stability and subcellular localization to RNA granules [6]. For example, heat-shock modulates the expression of various miRNAs in *Drosophila*, at both transcriptional and post-transcriptional levels, which in turn target stress-responsive genes [80]. Interestingly, a neuronal miRNA in *C. elegans*, *mir-71*, is required for appropriate response to heat stress [81], in line with the finding that the induction of major stress response mechanisms in worms, is orchestrated by specific neurons, probably through neuropeptide/neurotransmitter signalling [82,83].

Thus, it is possible that neuronal function of decapping complex in miRNA-mediated gene silencing elicits a coordinated response in the organism, through hormones or other signals, to regulate developmental progression and physiological functions at both normal and stress conditions. In accordance with this, we observed that *dcap-1* and *dcap-2* mutants have diminished mRNA levels of *ins-33*, a gene encoding an insulin/IGF-1-like peptide, which is a direct downstream target of LIN-14 during development [58]. INS-33 probably functions as a DAF-2/InsR agonist in somatic tissues to promote germline proliferation at the L3 stage [84], but also normal development at earlier stages: loss-of-function alleles of *ins-33* enhanced dauer entry of wild-type animals at 27°C and of *daf-2(e1368)* mutants at 22.5°C [85]. The latter information is consistent with our results on the increased dauer arrest of *daf-2(e1370)* and *daf-2 (e1368)* at 22°C upon loss of decapping genes (figure 1*a*). Thus, it seems that downregulation of *ins-33* owing to *dcap-1* mutation can enhance the activity of DAF-16, promoting dauer formation in Daf-c mutants. In line with this, we observed a slight increase of *sod-3* mRNA levels in *dcap-1* arrested animals, compared with N2 (electronic supplementary material, figure S1*d*). Of note, DAF-2/InsR and almost all of the 40 insulin/IGF-1-like peptides in *C. elegans* are expressed in neurons, acting both specifically and redundantly to modulate development, lifespan and stress responses in a cell non-autonomous manner [86]. In addition, several components of the IIS in many organisms are targets of miRNA regulation [35]. Intriguingly, the homologue of 5′–3′ exoribonuclease XRN-1 in *Drosophila* (known as Pacman) regulates the mRNA levels of the secreted insulin-like peptide Dilp8, which has been shown to coordinate tissue growth with developmental timing [87].

Overall, this work unveils a neuron-specific function of the mRNA decapping complex in providing robustness to developmental gene expression programmes, which is essential for survival under stressful conditions. These findings expand our perspectives on the functions of 5′–3′ mRNA decay factors during normal development and their influence on the regulation of miRNA targets upon environmental perturbations. Because these factors are evolutionarily conserved, we would expect them to play a similar functional role in proper regulation of developmental timing events, while also ensuring robustness in response to stress in higher organisms.

## 4. Material and methods

### 4.1. *Caenorhabditis elegans* strains and culture conditions

Standard methods of culturing and handling worms were used [88]. Worms were cultured on NGM plates seeded with *Escherichia coli* OP-50 or HT115 (DE3) for RNAi experiments. Wild-type Bristol N2 and some mutant strains were provided by the Caenorhabditis Genetics Center (CGC, University of Minnesota), which is supported by NIH Office of Research Infrastacture Programmes (P40 OD010440). Other mutant strains were provided by the Mitani Laboratory through the National Bio-Resource Project of the MEXT, Japan. All strains used in this study are shown in electronic supplementary material, table S1. All single mutants were crossed at least three times with N2 and double mutants were made by crossing the corresponding strains. Relevant mutations were tracked in F_2_ progeny either by PCR (see electronic supplementary material, table S2 for primers used) or phenotypic selection. *sid-1(pk3321)* mutation was tracked by selecting worms resistant to the embryonic lethality caused by feeding *rpl-19* dsRNA. *lin-14(n179)* mutation was tracked by phenotypic observation of animals at 25°C, where mutants exhibit various phenotypes (such as larval arrest precocious expression of adult alae at the L3 stage, vulval abnormalities and egg-laying defects) and subsequently verified by sequencing of a PCR product from the genomic locus encompassing the substitution (A-to-G) (using primers lin-14 FRW/lin-14 REV in electronic supplementary material, table S2). Transgenic animals were generated by microinjection of plasmid DNAs into the gonad of N2 or *dcap-1(tm3163)* young adults, using *rol-6(su1006)* as co-transformation marker [89]. Multiple lines were obtained for each genotype and screened for the representative expression pattern.

### 4.2. Phenotypic analysis

To determine the number of arrested worms, about 25–40 adult hermaphrodites of the various strains to be tested were allowed to lay eggs for a couple of hours (usually 1–3 h) at their standard growth temperature (15°C or 20°C) to obtain a population of synchronized embryos. After removal of the adults, eggs were transferred to assay temperatures (mentioned for each experiment in the text or the graphs) and were monitored for their developmental stage after 72 h (an adequate time interval for correct discrimination between normally growing and arrested worms). The mean percentage of dauer or arrested worms from at least three experiments ± standard deviation (s.d.) is presented in the graphs. For each experiment, all strains were assayed in parallel, with at least 100 animals dispersed in three to four plates per strain. The variability in the percentage of dauer or arrested larvae of a given strain is attributed to small differences in the assay temperature inside the incubator from experiment to experiment, which although small (less than 0.2°C) have significant effects on development at the environment of 27.5°C. When assessing the number of arrested worms in the transgenic strain BRF640 (for neuronal RNAi) fed with *lin-14* dsRNA, the percentage of transgenic worms was calculated in different plates at 20°C, using the rolling phenotype as a criterion (arrested worms at 27.5°C do not roll) and was used to calculate the number of non-transgenic arrested worms, which are resistant to RNAi by feeding and were therefore deducted from the experiment. To determine the growth rate of different strains, worms were synchronized with timed egg-laying (1–2 h) and the percentage of animals in each developmental stage was scored after 48 h at the indicated temperature.

### 4.3. Recovery and SDS-resistance assays

Recovery assays were used to score the capability of the N2 dauers or arrested *dcap-1* and *dcap-2* animals at 27.5°C to recover and continue their life cycle when placed back at 15°C. For this type of assay, adult worms were allowed to lay eggs at 20°C for a short period of time, and the eggs were transferred to 27.5°C for 3 days. N2 dauers or arrested decapping mutants were then picked and transferred to 15°C, and the percentage of recovered animals was counted 3–4 days later. In 1% SDS assay, *daf-2* dauers and arrested *dcap-1* or *dcap-2* worms were placed in 1% SDS solution in four-well plates and after 30 min the percentage of dead worms was counted [3]. Live worms were scored as dauers, whereas those that did not respond to touch and started to dissolve were scored as arrested.

### 4.4. High cholesterol and dafachronic acid assay

For high cholesterol test, worms of the indicated genotype were grown on NGM plates with 25 µg ml^−1^ cholesterol (5 µg ml^−1^ is the normal concentration) for one generation at 20°C. Synchronized eggs from adults raised in these plates were transferred at 27.5°C, and the percentage of arrested animals was scored after 3 days, in at least three plates for each strain. For sterol supplementation, (25S)-Δ7-DA (AdipoGen), dissolved in ethanol, was added to standard NGM plates seeded with OP-50 at a final concentration of 150 nM. Control plates were supplemented with the same volume of ethanol. Approximately 25 adults were allowed to lay eggs on DA or control plates at 20°C for a couple of hours and their eggs were transferred at 27.5°C. The percentage of arrested animals after 3 days was measured in three plates of each strain, in two independent experiments.

### 4.5. Constructs

RNAi plasmids were constructed by inserting gene-specific PCR product, amplified from genomic DNA using the appropriate primers (electronic supplementary material, table S2), into L4440 feeding vector (pPD129.36, Fire Kit, Addgene) [90]. For *gfp(RNAi)*, plasmid L4417 (pPD128.110, Fire Kit, Addgene) was used. Tissue-specific expression constructs of *dcap-1* were constructed as follows. A 4181-bp PCR fragment containing the *dcap-1* coding region fused with *gfp* and 1323-bp of *dcap-1* 3′UTR was obtained with primers DCAP-1/FRW and T7 XbaI from a *dcap-1::gfp* plasmid [39] and cloned in pBluescript KS(+) with *Bam*HI and *Xba*I to produce a promoterless *dcap-1::gfp* vector. All tissue-specific promoters were obtained by PCR from genomic DNA using the corresponding primers (electronic supplementary material, table S2) and cloned in the promoterless vector as *Pst*I/*Bam*HI or *Pst*I/*Pst*I fragments. In the case of *P_unc-119_::dcap-1* construct the promoter was cloned first with *Xho*I/*Pst*I in pBluescript KS(+) followed by the *dcap-1::gfp Bam*HI/*Xba*I fragment. The same *unc-119* promoter sequence was used for the construction of neuron-specific GFP::3′UTR*_lin-14_* and RFP::3′UTR*_unc-54_* constructs. For the RFP::3′UTR*_unc-54_* construct, the *rfp* sequence was amplified from pHb9::tagRFP (a gift from Dr Ivo Lieberam) and cloned in pPD95.77 (Fire Kit, Addgene) replacing *gfp* (*Age*I/*Eco*RI digest). For the GFP::3′UTR*_lin-14_* construct a 1761 bp of *lin-14* 3′UTR sequence was amplified from genomic DNA and cloned in pPD95.77 replacing the *unc-54* 3′UTR (*Eco*RI/*Eag*I digest). For the neuronal *sid-1* construct, a 6980 bp PCR fragment containing the coding region of *sid-1* with its native 3′UTR was amplified with primers sid-1/FRW and sid-1/REV from genomic DNA and cloned in pBluescript KS(+) vector with *Pst*I and *Bam*HI, followed by cloning of the same *unc-119* promoter fragment used for the *dcap-1* constructs with *Xho*I and *Pst*I. Neuronal GFP construct was created by digesting a 1864 bp fragment containing the *gfp* gene and the *unc-54* 3′UTR sequence from plasmid pPD95.77 with *Pst*I and *Spe*I and subsequent cloning in the pBluescript KS(+) vector containing the promoter of *unc-119*. Muscle GFP construct was created by excising a 2402 bp fragment containing the promoter of *myo-3* from plasmid L2534 (pPD96.52, Fire Kit, Addgene) with *Hind*III and *Xba*I and subcloning it in pPD95.77 vector.

### 4.6. Microscopy

For microscopic and fluorescent analysis, worms were monitored by mounting levamizol treated animals on 3% agarose pads on glass microscope slides. Images were captured by confocal microscopy using a Leica TCS SP5 II laser scanning confocal imaging system on a DM6000 CFS upright microscope and a 20× immersion objective, or by optical microscopy using a Leica DMRA2 upright microscope and 20×/63× objectives. When assessing the fluorescence of P*_sod-3_*::GFP or LIN-14::GFP in different genetic backgrounds, all animals were monitored under the same microscopy settings. LIN-14::GFP intensity was calculated separately for each worm and divided by the surface area of the animal. When assessing the GFP::3′UTR*_lin-14_*/RFP::3′UTR*_unc-54_* fluorescence microscopy settings were fine tuned for each strain and kept stable in all observed time points. GFP::3′UTR*_lin-14_* intensity was calculated for the whole image captured and then divided by the intensity of RFP::3′UTR*_unc-54_* for the whole image, to obtain the GFP/RFP ratio. All fluorescent images shown are two-dimensional maximal projections of z-stacks processed with Adobe Photoshop CS6. Fluorescence intensity of GFP and RFP reporters was measured using ImageJ 1.48t.

### 4.7. RNA isolation and quantitative reverse transcription PCR

For qRT-PCR, adults were allowed to lay eggs for 2–3 h at 20°C and plates were transferred at 25°C or 27.5°C for the proper amount of time in order to collect morphologically similar L2 larvae for RNA extraction. Total RNA was isolated from frozen worm pellets (200–300 worms) of the indicated genetic backgrounds and developmental stages, using Tri Reagent (Sigma-Aldrich), measured with Quant-iT RNA Assay Kit (Invitrogen) and reverse transcribed with iScript cDNA synthesis kit (Biorad, Hercules, CA). At least three populations of worms were harvested independently and analysed, in all experiments. For time-course qRT-PCR experiments, worms were cultured at 20°C and synchronized by standard hypochlorite treatment and L1 starvation at 25°C. Development was initiated the next day by transferring arrested L1 hatchlings on NGM plates seeded with OP-50 bacteria, at the temperatures of 25°C or 27.5°C. A lysate from 50 to 100 worms was produced for each sample with the treatment described by Ly *et al.* [91], and RNA was measured with Quant-iT RNA assay kit (Invitrogen) and reverse transcribed using Maxima H Minus First Strand cDNA Synthesis Kit with dsDNase (ThermoScientific). At least two populations of worms were harvested independently and analysed, in all experiments. Quantitative PCR was performed using the SsoFast EvaGreen supermix (BioRad) in the MJ MiniOpticon system (BioRad). The relative amounts of mRNA were determined using the comparative Ct method for quantification and gene expression data are presented as the fold change relative to control. qRT-PCR was performed in at least three independent biological samples, and each sample was independently normalized to endogenous reference *ama-1*. The mean ± standard deviation of at least three independent experiments is presented. The primer sequences used for qRT-PCR are shown in electronic supplementary material, table S2.

### 4.8. RNA interference

RNAi experiments were carried out by synchronizing worms on plates seeded with HT115(DE3) bacteria that express dsRNA for the indicated gene. For better interference results, animals were fed RNAi for two generations at 20°C before synchronous egg-laying and transfer at the indicated assay temperature. HT115 bacteria transformed with the relevant RNAi vectors were grown at 37°C in LB medium with ampicillin (50 µg ml^−1^) and tetracycline (10 µg ml^−1^). On the following day, fresh cultures with ampicillin were induced with 1 mM isopropylb-d-thiogalactopyranoside (IPTG) and seeded on RNAi plates, containing 1 mM IPTG [92]. Bacteria carrying the empty vector (pL4440) and treated likewise were used as control cultures (control (RNAi)).

### 4.9. Statistics

Statistical significance in all comparisons was determined using unpaired *t*-test analysis performed with GraphPad Prism v. 6.01 for Windows (GraphPad Software, La Jolla, USA, www.graphpad.com). Statistical significance was determined using unpaired *t*-test analysis (****p < 0.0001; ***p = 0.0001–0.001; **p = 0.001–0.01; *p = 0.01–0.05; n.s. indicates not significant, *p*-value ≥ 0.05).

## Supplementary Material

Supplementary Figures; Supplementary Table S1; Supplementary Table S2

## Data accessibility

The datasets supporting this article have been uploaded as part of the electronic supplementary material.
