# Supplementary material for: Neuronal function of the mRNA decapping complex determines survival of Caenorhabditis elegans at high temperature through temporal regulation of heterochronic gene expression
Source: Open Biol. 2017 Mar 1;7(3):160313. doi: 10.1098/rsob.160313 (PMC5376704; doi:10.1098/rsob.160313)
Supplement: Supplementary Figures; Supplementary Table S1; Supplementary Table S2 [file rsob160313supp1.pdf]

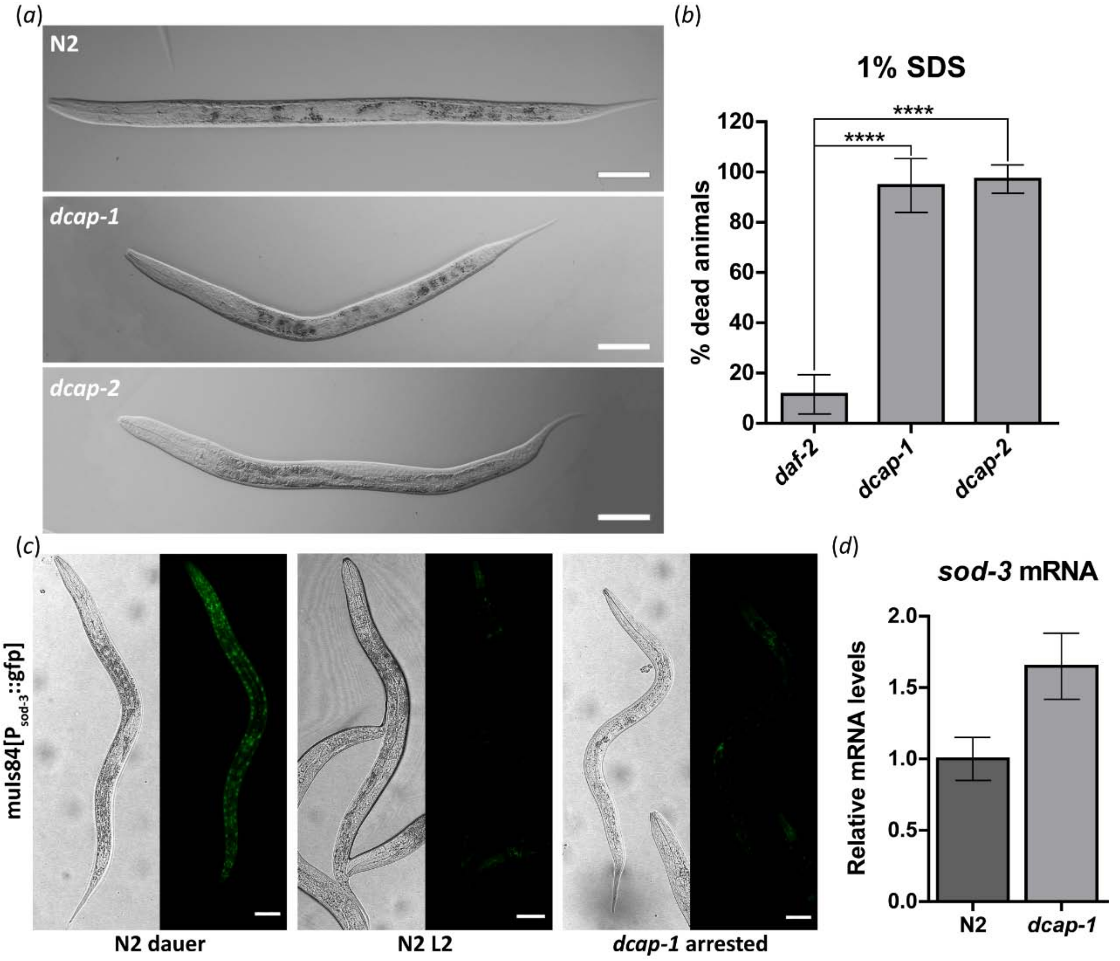

Figure S1. Decapping mutants arrest at L2-like stage when grown at 27.5°C. *(a)* Morphology of N2 worms arrested as dauers, and *dcap-1* or *dcap-2* mutants arrested in an early larval stage, morphologically similar to L2. Scale bar=50 µm *(b)* Percentage of dead animals after treatment with 1% SDS solution for 30 min. Worms were grown at 27.5°C and arrested animals were picked for treatment. *(c)* Representative confocal fluorescent images of worms carrying a transcriptional *sod-3::gfp* fluorescent reporter at 27.5°C. Scale bar=50 µm. *(d)* Relative mRNA levels of *sod-3* in N2 and *dcap-1* animals grown at 27.5°C for 24 hours post L1 arrest. Error bars represent standard deviation.

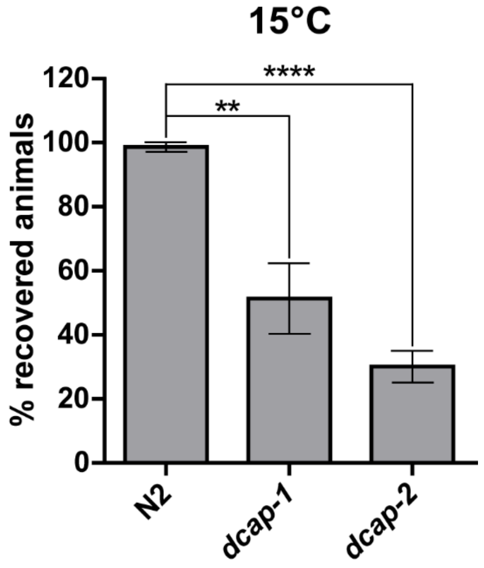

Figure S2. Decapping mutants do not fully resume normal development after transferring from 27.5°C to 15°C. Percentage of animals that recovered to adults when shifted to 15°C after developmental arrest at 27.5°C. The arrested N2 worms were dauers at 27.5°C and recovered to fertile adults at 15°C, in contrast to L2-like arrested *dcap-1* and *dcap-2* animals that resumed growth to nearly sterile adults. Error bars represent standard deviation.

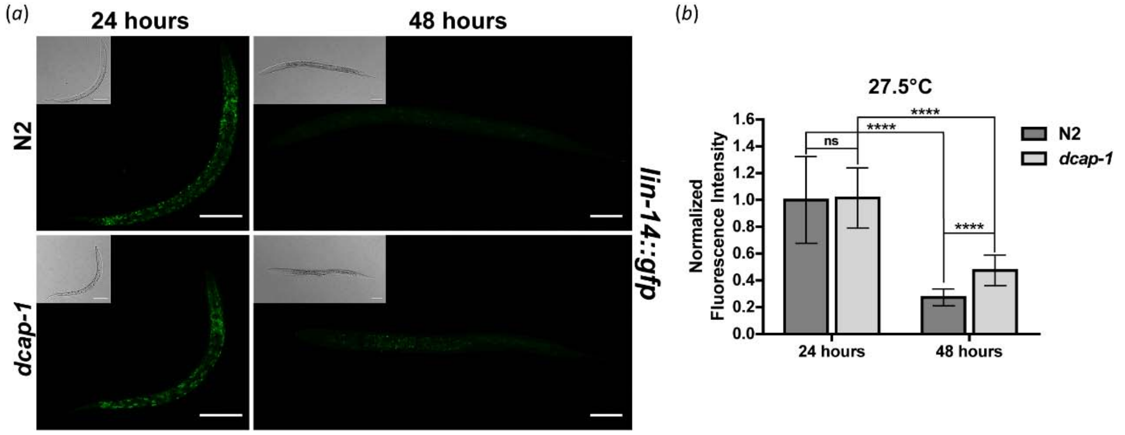

Figure S3. Accumulation of LIN-14 protein in *dcap-1* mutants during larval development at 27.5°C. *(a)* Representative confocal images of N2 and *dcap-1* mutant worms expressing a translational *lin-14::gfp* reporter 24 and 48 hours after egg-laying at 27.5°C. Scale bar=50 µm. *(b)* Quantified fluorescence of N2 and *dcap-1* mutants expressing a translational *lin-14::gfp* reporter 24 and 48 hours after egg laying at 27.5°C. Fluorescence was normalized to N2 worms at 24 hours. Error bars represent standard deviation.

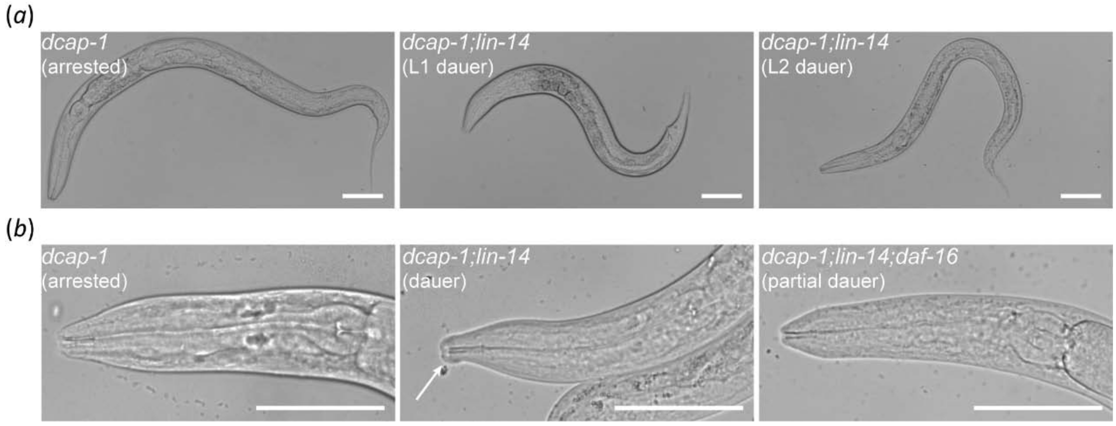

Figure S4. Morphology of *dcap-1;lin-14* and *dcap-1;lin-14;daf-16* arrested animals at 27.5°C. *(a)* Representative images of *dcap-1* and *dcap-1;lin-14* arrested larvae. Double mutants arrest either in the first or the second larval stage, forming L1 or L2 dauers respectively. *(b)* Representative images of *dcap-1, dcap-1;lin-14;* and *dcap1;lin-14;daf-16* arrested larvae showing the head region. The arrow points to the sealed mouth of *dcap-1;lin-14* mutants, a characteristic of dauer arrest that is absent in single *dcap-1* and triple *dcap1;lin-14;daf-16* mutants. Scale bar=50µm.

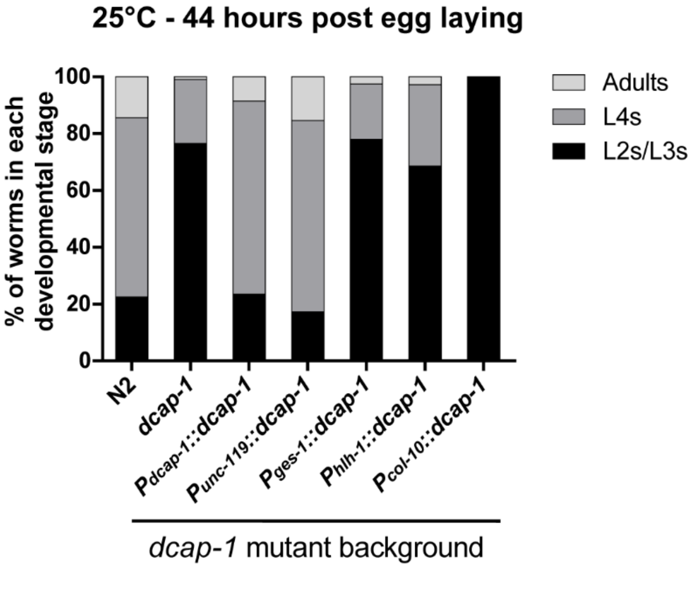

Figure S5. Neuron-specific *dcap-1* function influences developmental progression at normal growth temperatures. Distribution of *dcap-1* mutant animals carrying tissue-specific *dcap-1::gfp* transgenes among developmental stages, 44 hours post egg-laying at 25°C. N2 and *dcap-1* controls carry a *rol-6(su1006)* transgene (rollers).

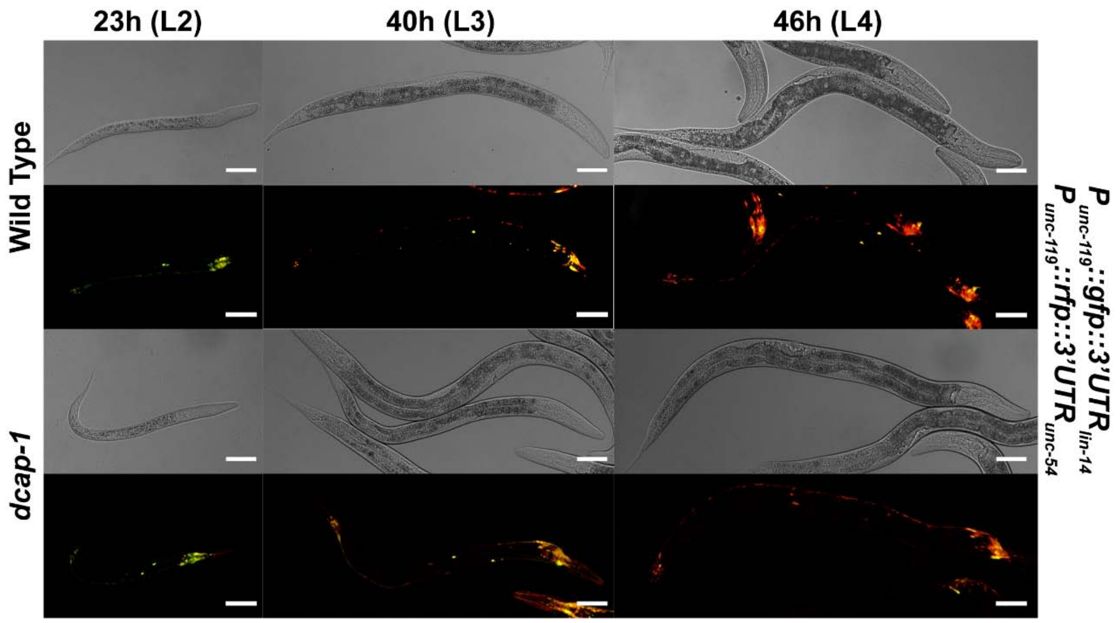

Figure S6. Misregulation of a *gfp* reporter bearing the *lin-14* 3'UTR in the nervous system of *dcap-1* mutants at the normal temperature of 25°C. Representative confocal images of WT and *dcap-1* worms carrying a *gfp::3'UTR*$_{lin\text{-}14}$ and an *rfp::3'UTR*$_{unc\text{-}54}$ transgene under the control of the pan-neuronal promoter *unc-119*, at time-points of 23, 40 and 46 hours post egg-laying at 25°C. Scale bar=50 µm.

Table S1. Strains used in this study

Strain	Genotype	Information	Reference
N2	N2 (Bristol)	Wild-type	CGC
BRF345	N2; *synEx31[rol-6(su1006)]*	Wild-type roller strain	This study
DR1572	*daf-2(1368)III*	Temperature sensitive Daf-c mutant of the insulin receptor *daf-2* (Class I allele)	CGC
CB1370	*daf-2(e1370)III*	Temperature sensitive Daf-c mutant of the insulin receptor *daf-2* (Class II allele)	CGC
BRF159	*dcap-1(tm3163)IV*	Mutant of the decapping regulatory subunit *dcap-1*	Rousakis *et al.*, 2014
BRF234	*dcap-2(ok2023)IV*	Mutant of the decapping catalytic subunit *dcap-2*	Rousakis *et al.*, 2014
BRF354	*dcap-1(tm3163)IV; synEx31[rol-6(su1006)]*	Cross of BRF159 males with BRF345 hermaphrodites	This study
BRF302	*daf-2(1368)III; dcap-1(tm3163)IV*	Mutant of the insulin receptor *daf-2* (Class I allele) and the decapping regulatory subunit *dcap-1*	Rousakis *et al.*, 2014
BRF306	*daf-2(e1368)III; dcap-2(ok2023)IV*	Mutant of the insulin receptor *daf-2* (Class I allele) and the decapping catalytic subunit *dcap-2*	Rousakis *et al.*, 2014
BRF637	*daf-2(e1370)III; dcap-1(tm3163)IV*	Cross of BRF159 males with CB1370 hermaphrodites	This study
CB1372	*daf-7(e1372)III*	Temperature sensitive Daf-c mutant of TGF-β ligand	CGC
BRF493	*daf-7(e1372)III; dcap-1(tm3163)IV*	Cross of BRF159 males with CB1372 hermaphrodites	This study
CF1553	*muIs84 [P_{sod-3}::GFP; rol-6(su1006)]*	Transcriptional reporter of *sod-3* in WT background	CGC
BRF131	*dcap-1(tm3163)IV; muIs84 [P_{sod-3}:: GFP; rol-6(su1006)]*	Cross of BRF159 males with CF1553 hermaphrodites	This study
CER60	*lsm-1(tm3585)II*	Mutant of the decapping co-activator *lsm-1*	Conres *et al.*, 2015
CF1038	*daf-16(mu86)I*	Daf-d mutant of the FOXO transcription factor *daf-16*	CGC
BRF412	*dcap-1(tm3163)IV; daf-16(mu86)I*	Cross of BRF159 males with CF1038 hermaphrodites	This study
MT355	*lin-14(n355)X*	Gain of function mutant of *lin-14* (Class I allele)	CGC
DR441	*lin-14(n179)X*	Temperature sensitive loss of function mutant of *lin-14* (Class II allele)	CGC
BRF656	*dcap-1(tm3163)IV; lin-14(n179)X*	Cross of BRF412 males with DR441 hermaphrodites (Selection for *daf-16* +/+)	This study
BRF657	*dcap-1(tm3163)IV; lin-14(n179)X; daf-16(mu86)I*	Cross of BRF412 males with DR441 hermaphrodites (Selection for *daf-16* -/-)	This study
CT21	*zaIs2[lin-14::GFP; rol-6(su1006)]*	Overexpression of translational fusion of *lin-14* with *gfp* in WT background	CGC
AA83	*daf-12(rh62rh157)X*	Daf-d mutant of *daf-12* (Class II allele)	CGC
BRF491	*dcap-1(tm3163)IV;daf-12(rh62rh157)X*	Cross of BRF159 males with AA83 hermaphrodites	This study
BRF259	*dcap-1(tm3163)IV; synEx190[P_{dcap-1}::dcap-1::gfp::3'UTR$_{dcap-1}$; rol-6(su1006)]*	Translational fusion of *dcap-1* with *gfp* in *dcap-1(tm3163)* background	Rousakis *et al.*, 2014
BRF250	*dcap-1(tm3163)IV; synEx293[$P_{unc-119}$::dcap-1::gfp::3'UTR$_{dcap-1}$; rol-6(su1006)]*	Neuronal specific expression of *dcap-1* under the control of *unc-119* promoter in *dcap-1(tm3163)* background	This study
BRF332	*dcap-1(tm3163)IV; synEx315[P_{osm-6}::dcap-1::gfp::3'UTR$_{dcap-1}$; rol-6(su1006)]*	Specific expression of *dcap-1* in ciliated sensory neurons under the control of *osm-6* promoter in *dcap-1(tm3163)* background	This study
BRF411	*dcap-1(tm3163)IV; synEx328[P_{ges-1}::dcap-1::gfp::3'UTR$_{dcap-1}$; rol-6(su1006)]*	Intestinal specific expression of *dcap-1* under the control of *ges-1* promoter in *dcap-1(tm3163)* background	This study
BRF356	*dcap-1(tm3163)IV; synEx326[P_{hlh-1}::dcap-1::gfp::3'UTR$_{dcap-1}$; rol-6(su1006)]*	Muscle specific expression of *dcap-1* under the control of *hlh-1* promoter in *dcap-1(tm3163)* background	This study
BRF478	*dcap-1(tm3163)IV; synEx364[P_{col-10}::dcap-1::gfp: 3'UTR$_{dcap-1}$; rol-6(su1006)]*	Hypodermal specific expression of *dcap-1* under the control of *col-10* promoter in *dcap-1(tm3163)* background	This study
NL3321	*sid-1(pk3321)V*	Mutant of the dsRNA transporter *sid-1.* Resistant to RNAi by feeding.	CGC
BRF645	*dcap-1(tm3163)IV; sid-1(pk3321)V*	Cross of BRF159 males with NL3321 hermaphrodites	This study
BRF640	*dcap-1(tm3163)IV; sid-1(pk3321)V;synEx288mn[$P_{unc-119}$::sid-1; $P_{unc-119}$::gfp; P_{myo-3}::gfp; rol-6(su1006)]*	Muscle and neuronal expression of gfp. Neuron-specific expression of *sid-1* in *dcap-1(tm3163)* and *sid-1(pk3321)* background. Neuron-specific RNAi by feeding.	This study
BRF542	*dcap-1(tm3163)IV; zaIs2[lin-14::GFP; rol-6(su1006)]*	Cross of BRF159 males with CT21 hermaphrodites	This study
VC446	*alg-1(gk214)X*	Mutant for Argonaut ortholog *alg-1*	CGC*
MH2385	*ain-1(ku322)X*	Mutant for GW182 homolog *ain-1*	CGC
BRF608	*synEx43742[$P_{unc-119}$::RFP::3'UTR$_{unc-54}$; $P_{unc-119}$::GFP::3'UTR$_{lin-14}$; rol-6(su1006)]*	Neuron specific expression of *rfp* fused with *unc-54* 3'UTR and *gfp* fused with *lin-14* 3'UTR in WT background	This study
BRF619	*dcap-1(tm3163)IV; synEx43742[$P_{unc-119}$::RFP::3'UTR$_{unc-54}$; $P_{unc-119}$::GFP::3'UTR$_{lin-14}$; rol-6(su1006)]*	Neuron specific expression of *rfp* fused with *unc-54* 3'UTR and *gfp* fused with *lin-14* 3'UTR in *dcap-1(tm3163)* background	This study

*C. elegans Reverse Genetics Core Facility at the University of British Columbia

Table 2. Primers used in this study

Primer	Sequence (5'-3')	Used for
DCAP-1/FRW	GGAAGGATCCATGAGCGACGCTAAAAAAAAG	Promoterless *dcap-1* construct
T7 XbaI	GGAATCTAGATAATACGACTCACTATAGGG	
unc-119p/FRW	GCCCTCGAGGCAATTGTTTTGTGCCAAGCTTCA	Cloning of *unc-119* promoter (*dcap-1* and *sid-1* constructs)
unc-119p/REV	GGCTGCAGATATGCTGTTGTAGCTGAAAATTTTGGG	
osm-6p/FRW	GTTAGTAATAGTAACGGGCTGATC	Cloning of *osm-6* promoter (*dcap-1* construct)
osm-6p/REV	CGGGATCCAGATGTATACTAATGAAGGTAATAGC	
ges-1p/FRW	ATGGCTGCAGTACCATTTTTCTTCGCG	Cloning of *ges-1* promoter (*dcap-1* construct)
ges-1p/Rev	AACTGCAGCTGAATTCAAAGATAAGATATG	
hlh-1p/FRW	CCCTGCAGGTTCTCGGTCTAACAGTGTC	Cloning of *hlh-1* promoter (*dcap-1* construct)
hlh-1p/REV	GGGGGATCCTTCTGGAAAATTATTGGAAAATTTGG	
col-10p/FRW	AAAACTGCAGAATGTTCACTTCCAATTTTCAGGTC	Cloning of *col-10* promoter (*dcap-1* construct)
col-10p/REV	AAAACTGCAGCTCTATGACTGAAAGCCAGGT	
RFP/FRW	GCTACCGGTAGAAAAAATGGTGTCTAAGGGCG	RFP::3'UTR_{unc-54} construct
RFP/REV	GAAGATCTATTAAGTTTGTGCCCCAGTTTG	
lin-14 3'UTR/FRW	CCTTTGAATTCACATCAGTCTCTTCACCCATC	GFP::3'UTR_{lin-14} construct
lin-14 3'UTR/REV	TTTCGGCCGGTAAGGTTCAGAGATGCATCC	
unc-119p/FRW2	TATCTGCAGGCAATTGTTTTGTGCCAAGCTTCA	Neuronal expression of GFP and RFP
unc-119p/REV2	GGATCTAGAATATGCTGTTGTAGCTGAAAATTTTGG	
sid-1/FRW	AACTGCAGCCTCATTTTTCCAGGTTCACAATG	Neuronal *sid-1* construct
sid-1/REV	CGCGGATCCCAGAAAGGTGTCATGGTCTAGTGG	
lin-14/FRW	GAAAAGCTTGCTCTCATGACGTCTCAAC	*lin-14* RNAi construct and *lin-14(n179)* genotyping
lin-14/REV	GAACTCGAGTTCGGATAAGATGGGTGAAG	
lin-28/FRW	AAACTGCAGCAAGGACTGAAAGGAAGCCG	*lin-28* RNAi construct
lin-28/REV	GAACTCGAGCCCTGAGAGTGCAATTTGAGG	
unc-13/FRW	CCTACTAGTGGAGAAGTTAGTGTTCAAGTTGAC	*unc-13* RNAi construct
unc-13/REV	CCACTCGAGATGCTGATGCAGCCATTACTG	
rpl-19/FRW	CAATCTCCGTCTGCAGAAAC	*rpl-19* RNAi constuct
rpl-19/REV	GCTCTAGACTTTCCAGCGATAACCTGC	
dcap-1/1	CGACGAAGCCTTTGCATG	*dcap-1(tm3163)* genotyping
dcap-1/2	GACGCCGATTGAGTGCAC	
daf-16/1	GGCTATTTCTCACTGTCTAC	*daf-16(mu86)* genotyping
daf-16/2	CGATTGAGTTCGGGGACTG	
daf-16/3	CAATGAGATTTATCAATGGTTCTC	
daf-12/1	GTTGTAGTAACTGTGATTGGTCC	*daf-12(rh157)* genotyping
daf-12/2	ATCTATCACTAGTCACTGGTC	
lin-14/RT1	TTTTAAGCTCACCCGCTGCAG	qRT-PCR
lin-14/RT2	TTCTGTCCATTACTATCCACGC	
lin-28/RT1	ACCAACACCTCGATACTTTGGC	qRT-PCR
lin-28/RT2	CTTCTACTTCTCCTGATACCGCAT	
ins-33/RT1	AGCCATGGACTTGGTGAAAC	qRT-PCR
ins-33/RT2	TTCGTCGTCGAAGAGGCTAT	
ama-1/RT1	GCTCCAAATGACCGAAATGT	qRT-PCR
ama-1/RT2	GCCCGGAGGAGATTAAACG	
